# Impact of Hyperoxia after Graft Reperfusion on Lactate Level and Outcomes in Adults Undergoing Orthotopic Liver Transplantation

**DOI:** 10.3390/jcm12082940

**Published:** 2023-04-18

**Authors:** Laurent Reydellet, Audrey Le Saux, Valery Blasco, Cyril Nafati, Karim Harti-Souab, Romain Armand, Ariane Lannelongue, Emilie Gregoire, Jean Hardwigsen, Jacques Albanese, Sophie Chopinet

**Affiliations:** 1 Department of Anaesthesia and Intensive Care, Hôpital la Timone, 13005 Marseille, France; 2 Department of Anaesthesia and Intensive Care, Carémeau Hospital, 30029 Nîmes, France; 3 Department of Digestive Surgery and Liver Transplantation, Hôpital la Timone, 13005 Marseille, France; 4 European Center for Medical Imaging Research CERIMED/LIIE, Aix-Marseille Université, 13385 Marseille, France; 5 École de Médecine, Faculté des Sciences Médicales et Paramédicales, Aix-Marseille Université, 27 Boulevard Jean Moulin, 13385 Marseille, France

**Keywords:** liver transplantation, hyperoxia, ischemia–reperfusion injury, lactate level

## Abstract

Background: Hyperoxia is common during liver transplantation (LT), without being supported by any guidelines. Recent studies have shown the potential deleterious effect of hyperoxia in similar models of ischemia–reperfusion. Hyperoxia after graft reperfusion during orthotopic LT could increase lactate levels and worsen patient outcomes. Methods: We conducted a retrospective and monocentric pilot study. All adult patients who underwent LT from 26 July 2013 to 26 December 2017 were considered for inclusion. Patients were classified into two groups according to oxygen levels before graft reperfusion: the hyperoxic group (PaO_2_ > 200 mmHg) and the nonhyperoxic group (PaO_2_ < 200 mmHg). The primary endpoint was arterial lactatemia 15 min after graft revascularization. Secondary endpoints included postoperative clinical outcomes and laboratory data. Results: A total of 222 liver transplant recipients were included. Arterial lactatemia after graft revascularization was significantly higher in the hyperoxic group (6.03 ± 4 mmol/L) than in the nonhyperoxic group (4.81 ± 2 mmol/L), *p* < 0.01. The postoperative hepatic cytolysis peak, duration of mechanical ventilation and duration of ileus were significantly increased in the hyperoxic group. Conclusions: In the hyperoxic group, the arterial lactatemia, the hepatic cytolysis peak, the mechanical ventilation and the postoperative ileus were higher than in the nonhyperoxic group, suggesting that hyperoxia worsens short-term outcomes and could lead to increase ischemia–reperfusion injury after liver transplantation. A multicenter prospective study should be performed to confirm these results.

## 1. Introduction

Liver transplantation has emerged as the reference treatment for advanced cirrhosis, localized hepatocellular carcinoma and fulminant forms of acute liver failure. Since first being performed in Denver in 1963, it has revolutionized the prognosis of these patients through improvements in surgical techniques, anesthetic and intensive care management as well as the optimization of immunosuppressive therapies. In 2019, before the COVID-19 pandemic, 1356 liver transplantation were performed, bringing the transplant rate to 20.5 per million inhabitants, the highest rate ever reached [1].

During liver transplantation, graft revascularization is a critical operative time with a significant risk of ischemia–reperfusion (IR) injury [2]. This syndrome is characterized by the occurrence of hemodynamic instability due to the massive release of free oxygen radicals, endotoxins and inflammatory cytokines. After a cold ischemia phase, the graft is subjected to a warm ischemia phase during the implantation. These two phases are responsible for cell damage and have already been studied in previous studies. After unclamping the portal vein, reperfusion is the last step that amplifies the induced lesions. They are the main causes of primary graft dysfunction [3]. The overproduction of reactive oxygen species is one of the main mechanisms described in IR syndrome.

The administration of supplemental oxygen is a cornerstone to overcoming hypoxia in critical ill patients admitted to the ICU for cardiac arrest, traumatic injury, and post-cardiac injury. Although oxygen therapy is a life-saving therapy, excessive oxygen levels can have adverse effects on patients and worsen their condition. Hyperoxia may affect biological systems such as ROS overproduction due to excessive production of cytokine and alteration of antioxidant enzymes. In animal models, it appears to be increased by hyperoxia with increased induced lesions [4,5]. Hyperoxia is defined as a supraphysiological supply of oxygen. In humans, direct pulmonary toxicity is probably the most well-known adverse consequence of hyperoxia. However, clinical studies regarding the impact of hyperoxia on critical ill patients have shown conflicting results [6,7].

During ventilator-assisted general anesthesia, altered oxygenation is common [8], leading practitioners to maintain a high fraction of inspired oxygen (FiO_2_) in high-risk situations such as liver transplantation. This common practice is not supported by any guidelines [9].

In recent years, many studies have investigated the possible adverse consequences of hyperoxia. In particular, the PROXI trial [10] found an association between the perioperative administration of a high FiO_2_ and the increase in long-term mortality. In addition, studies of similar models of IR, such as myocardial infarction or stroke, have shown the deleterious potential of massive oxygen delivery to lesions following organ revascularization [11,12].

The definition of hyperoxia varies among previous studies. Some authors use FiO_2_ to define hyperoxia, and in other studies, PaO_2_ thresholds vary from 120 mmHg to 300 mmHg to define hyperoxia. However, in several meta-analyses on the impact of hyperoxia on patients admitted to the ICU, the threshold of 200 mmHg PaO_2_ was the most frequently used. Therefore, we have chosen the threshold of 200 mmHg in accordance with previous studies [13,14].

The perioperative period is a critical period during liver transplantation and impacts short- and long-term outcomes, as previously described. Indeed, the perioperative period impacts renal function and short- and long-term outcomes due to perioperative outcomes: hemodynamic instability, immunosuppressive induction and transfusion, which can lead to impaired injury. In a previous study, we described the factors that impact renal impairment at one year: MELD-score, blood transfusion > 3 Red blood units, serum lactate levels at ICU ≥ 2.5 mmol/L, ICU stay ≥ 5 days and norepinephrine dose. These results highlight the major impact of perioperative outcomes on long-term outcomes. This study demonstrated the impact of post-liver transplantation arterial lactate levels on postoperative outcomes [15].

Hence, the impact of hyperoxia on lactate levels seems particularly relevant in liver transplantation.

To our knowledge, this is the first study that assesses the impact of hyperoxia in liver transplantation, examining the deleterious potential of hyperoxia. We therefore conducted a retrospective study to evaluate its impact after graft reperfusion during orthotopic liver transplantation in adults.

## 2. Materials and Methods

### 2.1. Study Design and Population

This is an observational, retrospective, monocentric study that was conducted in the Department of Anesthesia and Liver Pathologies Intensive Care Unit (ICU) of the University Hospital Center of la Timone in Marseille.

All patients aged 18 years and older who received an orthotopic liver transplant between 26 July 2013 and 26 December 2017 were considered for inclusion. Only patients who had an incomplete medical record, making the analysis of the primary endpoint impossible, were excluded.

Intraoperative anesthetic management of transplant patients was realized according to the service protocol. Induction, maintenance of anesthesia, equipment, patient monitoring, transfusion strategy and hemodynamic management were standardized.

Surgically, transplantation was conducted as frequently as possible with lateral inferior vena cava clamping and with the first vein unclamped being the portal vein, this being the most common method in France.

During the data collection period, the operative and anesthetic protocols were the same for all patients included in this study.

### 2.2. Data Collection

The entirety of the data was collected from the patient’s paper or electronic medical records and recorded in a standardized file in the form of a Microsoft Excel^®^ spreadsheet.

The variables collected for transplant patients (recipients) were: demographic characteristics (age, sex), anthropomorphic characteristics (height, weight, body mass index), preoperative assessment (hemoglobin, platelets, prothrombin ratio (PR), factor V, aspartate transaminase (AST), alanine transaminase (ALT), alkaline phosphatase (AP), gamma-glutamyl transpeptidase (GGT), bilirubin, creatinine, albumin), CHILD score, MELD score (Model for End-stage Liver Disease) and liver transplantation indications.

Demographic (age, sex) and anthropomorphic (height, weight, BMI) characteristics of the donor, as well as their preoperative blood assessment (PR, bilirubin, AST, ALT, AP, GGT, natremia), were also collected.

Intraoperative data, such as transfusion of labile blood products, cold ischemia time, anhepatic time and operating time, were also collected.

### 2.3. Definition and Study Endpoints

Arterial blood gases, routinely performed 15 min after vascular unclamping, were used to classify transplant patients into two groups: a hyperoxic group defined by a partial arterial oxygen pressure (PaO_2_) greater than 200 mmHg and a non hyperoxic group defined by a PaO_2_ of less than 200 mmHg.

The primary endpoint was arterial lactatemia 15 min after vascular unclamping, a time which corresponds with the hepatic graft revascularization phase.

The secondary endpoints were arterial lactatemia at the end of the liver transplantation, the hepatic cytolysis peak within the first 7 postoperative days, norepinephrine dosage on arrival in the intensive care unit, duration of mechanical ventilation, duration of ileus (defined as the number of days without stool), postoperative complications, length of stay in the intensive care unit, total duration of hospitalization, death in intensive care unit and death at 1 year.

Postoperative complications included the occurrence of acute renal failure (defined as an increase of ≥26.5 μmol/L of creatinine or 1.5 times preoperative plasma creatinine according to the KDIGO classification) with or without recourse to extrarenal blood filtering, hemorrhagic complications (defined by the need for a transfusion >2 red blood cell units and/or surgical reintervention), cardiovascular complications (defined by the occurrence of a cardiac rhythm disorder, acute pulmonary edema, cardiogenic shock or cardiac arrest), respiratory failure (defined by the occurrence of ventilatory disorders or pneumonia) and the occurrence of sepsis (defined by the occurrence of a systemic inflammatory response syndrome with a suspected or proven infection).

Early allograft dysfunction was defined according to the Olthoff criteria [16].

### 2.4. Statistical Analysis

Data were entered in a Microsoft^®^ Office Excel 2013 spreadsheet and analyzed by SPSS.v.20^®^ software (IBM Corp., Armonk, NY, USA). Quantitative data are presented as a mean and standard deviation. They were compared by the student *t*-test. Qualitative data are expressed as absolute values and percentages. They were compared by a Chi2 test. The statistical significance threshold was 5% (*p* < 0.05).

### 2.5. Ethical Validation

The study was registered at the National Commission of Informatics and Liberty. This study was approved by the ethics committee of Aix Marseille university and registered under the number 2023-03-08.

## 3. Results

### 3.1. Study Population

From 26 July 2013 to 26 December 2017, 262 adult patients underwent orthotopic liver transplantation and were considered for inclusion. Of these, 40 patients were excluded due to missing data on arterial blood gas measurement for analysis of the primary endpoint.

**Figure 1:**
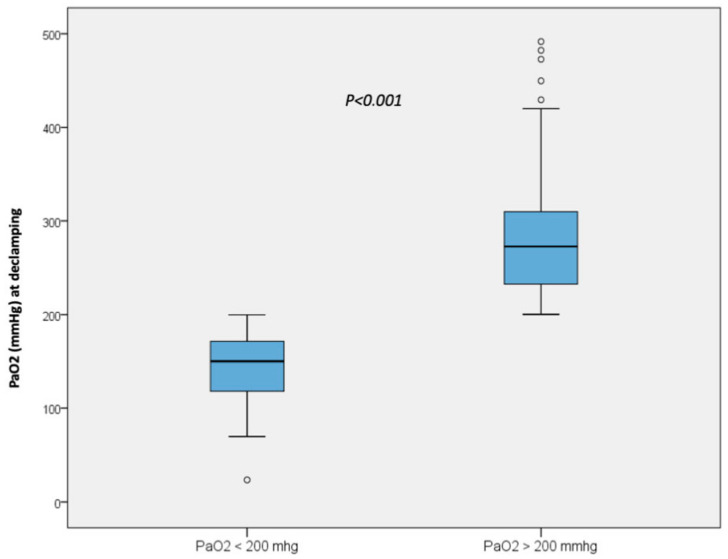
PaO_2_ at declamping in the non-hyperoxic group and in the hyperoxic group.

A total of 222 patients were included for analysis with 118 patients in the nonhyperoxic group (PaO_2_ < 200 mmHg) and 104 patients in the hyperoxic group (PaO_2_ > 200 mmHg) (Figure 1).

The groups were comparable across all demographic criteria, anthropomorphic criteria, liver transplantation indications and preoperative laboratory data assessment (Table 1).

Recipients were predominantly male with, respectively, 78% and 73% in the nonhyperoxic and hyperoxic groups (*p* = 0.35). The mean age of recipients was similar in both groups: 53 ± 10 years in the nonhyperoxic group and 53 ± 11 years in the hyperoxic group (*p* = 0.74). The mean MELD score was 21 ± 10 in the nonhyperoxic group and 20 ± 11 in the hyperoxic group (*p* = 0.35).

The characteristics of the donors are detailed in Table 2. The mean age in the nonhyperoxic group was 57 years ± 16 vs. 53 years ± 17 in the hyperoxic group (*p* = 0.03). There were no differences between the two groups regarding biological assessment.

Intraoperative data does not present significant differences between the two groups. Operative time was 304 min ± 68 in the nonhyperoxic group and 294 ± 63 min in the hyperoxic group (*p* = 0.26). The cold ischemia time was 456 ± 125 min in the non hyperoxic group vs. 462 ± 123 min in the hyperoxic group (*p* = 0.70). During the operative period, transfusion of labile blood products was similar between the two groups (Table 3).

### 3.2. Primary Endpoint

**Figure 2:**
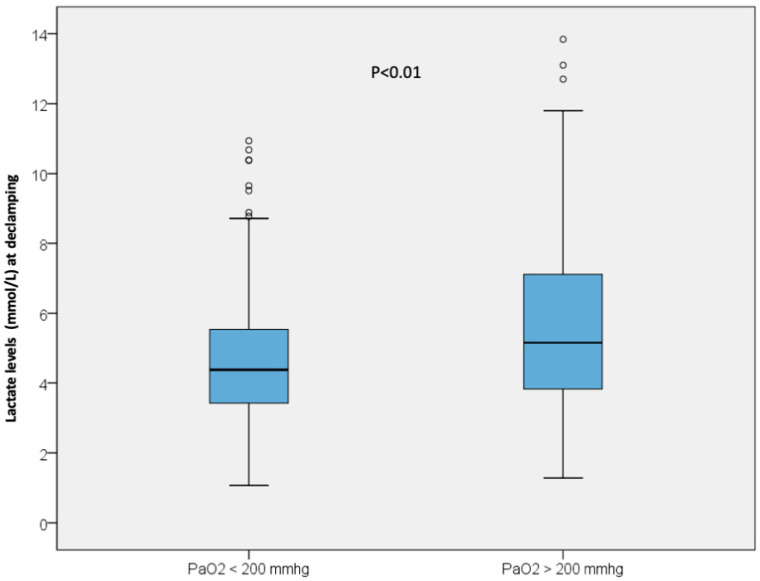
Lactate level at 15 min after graft reperfusion in the non-hyperoxic group and in the hyperoxic group.

Arterial lactatemia at 15 min after graft reperfusion was significantly higher in the hyperoxic group (6.03 ± 4 mmol/L) than in the nonhyperoxic group (4.81 ± 2 mmol/L), *p* < 0.01 (Figure 2).

### 3.3. Secondary Endpoints

Arterial lactatemia at the end of the liver transplantation was significantly higher in the hyperoxic group (4.22 ± 3.8 mmol/L) than in the nonhyperoxic group (3.25 ± 2.1 mmol/L), *p* = 0.02. The hepatic cytolysis peak within 7 postoperative days was significantly higher in the hyperoxic group, with, respectively, 1570 ± 2457 IU/L vs. 1015 ± 1540 IU/L (*p* = 0.04) for AST and 988 ± 1118 IU/L vs. 699 ± 856 IU/L (*p* = 0.03) for ALT. There were more early allograft dysfunctions in the hyperoxic group than in the nonhyperoxic group, at 42% vs. 26%, respectively (*p* = 0.02). There was no statistically significant difference regarding the other biological parameters: PT, factor V and bilirubinemia at day 7. Norepinephrine levels at admission to the ICU were similar in both groups. The duration of mechanical ventilation was statistically longer in the hyperoxic group (24 ± 49 h), than in the nonhyperoxic group (11 ± 13 h), *p* < 0.01. Postoperative ileus was significantly longer in the hyperoxic group (4.9 ± 1.9 days) than in the nonhyperoxic group (4.3 ± 1.6 days), *p* = 0.03.

There was no statistically significant difference in ICU length of stay (11.6 ± 14 vs. 20.6 ± 48 days), *p* = 0.05. Total hospital stays, ICU and 1-year mortality, and incidence of postoperative complications were not statistically different between the two groups. The incidence of acute kidney injury was higher in the hyperoxic group than in the nonhyperoxic group, 42% vs. 30% (*p* = 0.06), but the difference was not statistically significant.

Perioperative and postoperative outcomes are presented in Table 4.

## 4. Discussion

The organ shortage has led us, in recent years, to broaden the eligibility criteria for donors. During liver transplantation, perioperative period is critical, and several factors, such as lactates levels and length of ICU stay, have been identified to impact the long-term outcome [15]. The prevention of IR syndrome in these situations is a major concern since its occurrence implies significant morbidity and mortality in recipients [17]. The identification of modifiable risk factors is critical to improve short- and long-term outcomes after LT.

The main objective of this study was to assess the potential impact of intraoperative hyperoxia in adults’ liver transplantation. Our results indicate that arterial lactatemia 15 min after liver graft reperfusion is higher in patients exposed to intraoperative hyperoxia than in patients in the nonhyperoxic group. To the best of our knowledge, this is the first study to show an increase in hyperoxia-related lactates 15 min after reperfusion in liver transplantation. The second endpoint of this study has shown an increase of hepatic cytolysis peak and EAD [14], longer mechanical ventilation and postoperative ileus in the hyperoxic group, despite no difference found in ICU stay, postoperative complications or 1-year mortality between the two groups.

The arterial blood lactate level is already known to be increased in IR situations. The study by Theodoraki et al. [18] showed a significant increase in lactate levels 50 min after hepatic reperfusion in the context of hepatectomies performed under vascular exclusion. In this study, the increase in arterial lactatemia was significantly correlated with the transhepatic gradient of lactates, suggesting lactate production by the liver.

Indeed, the elevation of the arterial lactate level could be related to both an overproduction of lactates due to cellular hypoxia and a lack of clearance during the anhepatic stage. Multifactorial mechanisms are involved in the rise of lactate levels during liver graft reperfusion. During hepatic ischemia, the metabolism is changed from an aerobic to anaerobic form due to cellular hypoxia. The reserves of adenosine triphosphate are depleted, leading to an acceleration of glycolysis [19]. If ischemia is prolonged, intracellular acidosis eventually inactivates anaerobic glycolysis and causes ATP-dependent pumps to shut down. The accumulation of the end products of the different metabolic pathways and uncompensated ionic disorders lead to loss of membrane polarity, cell swelling by water entry and disruption of the cytoskeleton. Irreversible cellular lesions, mainly by necrosis, will then appear.

There is a paradox related to oxygen at the reperfusion phase; indeed, administration of supplemental oxygen is a cornerstone to overcoming hypoxia, despite ischemia–reperfusion injury. After a prolonged hypoxia time leading to cellular apoptosis and hepatocyte necrosis, the sudden influx of oxygen during reperfusion leads to an inflammatory chain reaction and especially to the overproduction of ROS, worsening reperfusion injury. The capacities of cellular antioxidant systems are rapidly saturated, which will aggravate the effects of ROS. This defines oxidative stress. This phenomenon is even more intense by the increase of oxygen levels [20]. The inflammatory chain reaction resulting from IR syndrome could also cause serious microcirculatory disorders [21]. Especially in the study by Theodoraki et al. [18], the authors have shown a decrease in the transhepatic gradient of PaO_2_ after reperfusion, suggesting a difficulty of extraction and use of oxygen by the hepatic parenchyma. Other mechanisms, such as increased hepatic glucose, mediated by high levels of cytokines, could also be implicated [22,23]. In vitro, endothelial cell injury results in the inhibition of pyruvate dehydrogenase, leading to the accumulation of pyruvate [24]. Thus, the majority of pyruvate produced by glycolysis is diverted from an aerobic metabolism and, consequently, converted into lactate-by-lactate dehydrogenase [25]. Thus, the increase in the lactate level is related to the extent of reperfusion injury induced after reperfusion of the liver graft.

In the literature, the elevation of lactate is an independent risk factor of morbidity and mortality [23,26,27,28]. In addition, the elevation of the arterial lactate level is correlated with the occurrence of hepatic and renal dysfunction and to mortality during hepatic resection [15,29].

In our study, the postoperative hepatic cytolysis peak was significantly higher in the hyperoxic group, indicating the aggravation of reperfusion injury. Early allograft dysfunction was also more frequent in the hyperoxic group. In mice, liver lesions after reperfusion are significantly aggravated by hyperoxia [30].

This hypothesis also seems to be confirmed by a significantly longer duration of mechanical ventilation and longer postoperative ileus in the hyperoxic group, which is consistent with data from previous studies evaluating the impact of hyperoxia in critically ill patients. Indeed, the duration of mechanical ventilation seems to be prolonged in patients with severe IR syndrome, as found in the Hilmi et al. study [31]. In addition, in liver transplantation from living donors, lactatemia ±8.2 mmol/L was identified as an independent predictor of late mechanical ventilation weaning [32]. In addition, prolonged mechanical ventilation is associated with increased mortality [33]. In this study, all consecutive orthotopic liver transplants were included. Only donors after brain death were included in this study. Living donors are very rare in France, and none were included in this study. No donors after cardiac arrest were included in this study. The initial French experience regarding liver transplantation after cardiac arrest began recently [34], and the results were recently reported. In the French protocol for donors after cardiac arrest, normothermic regional perfusion is systematically used. The initial experience did not show any difference in postoperative outcome between donors after cardiac arrest and brain-dead donors [35]. It would be interesting to evaluate the impact of hyperoxia in patients transplanted from donors after cardiac arrest in further studies.

Postoperative ileus persisted for a longer time in the hyperoxic group than in the nonhyperoxic group. Compared to other abdominal surgeries, liver transplantation has a higher incidence of postoperative ileus and delayed recovery of gastrointestinal function [36]. Its origin is multifactorial and depends on previous abdominal surgery and the difficulty of the surgery, but a part may be due to ischemia–reperfusion lesions with an important role of splanchnic and hepatic ischemia–reperfusion phenomena. The lengthening of postoperative ileus is a recognized factor of poor prognosis after abdominal surgery. It is responsible for increased postoperative complications and duration of hospital stay [37].

Although the difference was not statistically significant (*p* = 0.06), the incidence of postoperative renal failure was higher in the hyperoxic group than in the nonhyperoxic group. In the study by Paugam et al. [38], liver transplant recipients with reperfusion syndrome were more likely to develop severe renal failure. Hemodynamic changes and direct necrosis of tubular cells are implicated in the pathophysiology of renal failure [39]. On the other hand, hemorrhagic complications were twice as frequent among patients of the hyperoxic group. This result is not statistically significant (*p* = 0.07), although the impact of ischemia–reperfusion on coagulopathy and the use of blood transfusion is widely described in the medical literature [31,40].

To our knowledge, there are no studies that have investigated the potential deleterious effect of hyperoxia during graft reperfusion in organ transplantation in humans. Only animal studies suggest that a high oxygen-inspired fraction may increase graft reperfusion injury [30,41].

However, the medical literature has been largely concerned with the impact of hyperoxia in similar models of ischemia–reperfusion in humans. Although the results are heterogeneous, the latest studies induce clinicians to reflect on the potential consequences of a massive supply of oxygen.

The clinical trial AVOID [11] notably showed an increase in the size of myocardial ischemic lesions at 6 months of infarction in patients who received a routine oxygen administration. These results are also found in the study by Guensch et al. [42]. During cardiopulmonary arrest, hyperoxia is associated with an increase in mortality [43,44,45]. It could also be responsible for a worsening of the neurological sequelae that result from a marked cerebral vasoconstriction [46,47]. Similarly, in the Rincon et al. [12] study, hyperoxia was independently associated with mortality in patients admitted to intensive care units for cerebral infarction.

In patients ventilated in the intensive care, the feasibility and safety of a restrictive strategy of oxygenation levels has already been studied. The pilot study by Suzuki et al. [48] showed that this was a safe practice since it did not result in a significant difference in the outcome of patients and even showed a tendency to decrease respiratory failure and lower lactate levels.

The result of our study suggests a change in practices regarding the use of high FiO_2_ levels during the reperfusion period in liver transplantation. The titration of FiO_2_ would avoid hyperoxia and its potential deleterious effect on the worsening of lesions induced by ischemia–reperfusion.

There are several limits in this study. First of all, this is a monocentric, observational, retrospective study that has inherent limitations in collecting data retrospectively. Missing data were noted during this work (15.2%). The number of patients included is substantial since 222 liver transplant patients during the study period were analyzed. However, this size sample is probably insufficient to show a statistically significant difference in the incidence of postoperative complications or mortality. Moreover, our population seems representative of that of the other centers of liver transplantation in France, composed of 13 medical–surgical teams with an exclusively adult orientation. In our study, the mortality at 1 year was 11.7%, which is comparable to that observed in France for the period 2014–2016 (11.6%) [1].

The second limit is represented by the choice of the hyperoxia threshold. The definition of hyperoxia is variable in studies that explore its effects, which may partly explain the heterogeneity of their result. It can be defined as a supraphysiological increase in arterial oxygen partial pressure (PaO_2_). The elevation of dissolved oxygen in the bloodstream represents the main effect of the increase of the inspired fraction under normal pH and temperature conditions. It is thus responsible for the overproduction of reactive oxygen species [4,49].

The available data did not allow us to determine the exposure time to hyperoxia. However, the overproduction of free radicals seems to occur mainly during the first moments of reperfusion. Early hyperoxia would thus be the main mechanism of worsening lesions secondary to ischemia–reperfusion [50].

In addition, we did not take into account the degree of steatosis of hepatic grafts. Steatosis livers are more sensitive to ischemia–reperfusion, which is a potential confounding factor. Nevertheless, donor characteristics were similar in both groups besides age, with significantly younger donors in the hyperoxic group [51].

## 5. Conclusions

Our study suggests that arterial lactatemia after liver graft revascularization is higher in transplant patients exposed to hyperoxia. This result could be the consequence of worsening ischemia–reperfusion injury by hyperoxia. The increase in the hepatic cytolysis peak and the durations of mechanical ventilation and postoperative ileus seem to support this hypothesis. However, these results have to be confirmed by a larger prospective study.

## Figures and Tables

**Table 1:** Characteristics of recipients.

	Group PaO_2_ < 200 mmHg (*n* = 118)	Group PaO_2_ > 200 mmHg (*n* = 104)	*p*
Age (years)	53 ± 10	53 ± 11	0.74
Male	93 (78%)	76 (73%)	0.35
Size (cm)	171 ± 18	172 ± 10	0.60
Weight (kg)	76 ± 16	74 ± 15	0.20
BMI (kg/m^2^)	26 ± 4.3	25 ± 4.6	0.22
Child-Pugh score	10 ± 3	9 ± 3	0.09
MELD score	21 ± 10	20 ± 11	0.35
PR (%) < 70	48 ± 22	53 ± 25	0.14
Factor V (IU/L)	0.52 ± 0.32	0.56 ± 0.33	0.35
Total bilirubin (µmol/L) < 17	138 ± 159	119 ± 152	0.36
AST (IU/L) < 34	194 ± 696	216 ± 767	0.82
ALT (IU/L) < 34	136 ± 445	197 ± 760	0.46
GGT (IU/L) < 45	130 ± 218	158 ± 197	0.34
AP (IU/L) < 70	174 ± 143	172 ± 148	0.95
Hemoglobin (g/dL)	10.7 ± 2.4	10.9 ± 2.3	0.38
Platelets (G/L) (150–250)	100 ± 68	104 ± 67	0.67
Serum creatinine (µmol/L)	107 ± 78	109 ± 82	0.87
Albumin (g/L) (35–50)	32 ± 7	33 ± 7	0.50
Etiologies:			
Cirrhosis	95 (80%)	77 (74%)	0.25
Alcoholic cirrhosis	40 (34%)	24 (23%)	0.08
Viral cirrhosis	21 (18%)	23 (22%)	0.42
NASH cirrhosis	10 (8%)	5 (5%)	0.27
Combined cirrhosis	13 (11%)	14 (13%)	0.58
AAH	2 (1.7%)	2 (1.9%)	0.90
HCC	34 (29%)	33 (32%)	0.63
Hepato-biliary pathologies	5 (4.2%)	5 (4.8%)	0.83
Fulminant hepatitis	5 (4.2%)	6 (5.8%)	0.60
Re-transplants	7 (6%)	10 (9.6%)	0.30
Others	17 (14%)	22 (21%)	0.19

Data are expressed as mean ± standard deviation or absolute value (percent). PaO_2_, oxygen partial arterial pressure; PR, prothrombine ratio; AST, aspartate amino-transaminase; ALT, alanine-amino transaminase; GGT, gamma glutamyl transferase; AP, alkaline phosphatase; NASH, nonalcoholic steatosis hepatitis; AAH, Acute Alcoholic Hepatitis; HCC, hepatocellular carcinoma.

**Table 2:** Characteristics of donors.

	Group PaO_2_ < 200 mmHg (*n* = 118)	Group PaO_2_ > 200 mmHg (*n* = 104)	*p*
Age (years)	57 ± 16	53 ± 17	0.03
Size (cm)	169 ± 9	170 ± 9	0.66
Weight (kg)	75 ± 14	74 ± 15	0.60
BMI (kg/m^2^)	26 ± 5	26 ± 5	0.44
PR (%)	68 ± 15	70 ± 16	0.26
Total bilirubin (µmol/L) < 17	12 ± 9	11 ± 7	0.53
AST (IU/L) < 34	81 ± 190	61 ± 70	0.31
ALT (IU/L) < 34	66 ± 144	49 ± 53	0.24
GGT (IU/L) < 45	49 ± 57	48 ± 49	0.94
AP (IU/L) < 70	75 ± 40	70 ± 32	0.34
Natremia (mmol/L) < 133	147 ± 8	147 ± 8	0.99

Data are expressed as mean ± standard deviation. BMI, body mass index; PR, prothrombin ratio; AST, aspartate amino-transaminase; ALAT, alanine amino-transaminase; GGT, gamma glutamyl transferase; AP, alkaline phosphatase.

**Table 3:** Intraoperative data.

	Group PaO_2_ < 200 mmHg (*n* = 118)	Group PaO_2_ > 200 mmHg (*n* = 104)	*p*
PaO_2_ after reperfusion (mmHg)	141 ± 39	281 ± 67	<0.01
Duration of surgery (minutes)	304 ± 68	294 ± 63	0.26
Duration of cold ischemia (minutes)	456 ± 125	462 ± 123	0.70
Anhepatic duration (minutes)	70 ± 23	71 ± 23	0.67
Transfusion:			
RBC (units)	2.7 ± 3.8	2.9 ± 4.4	0.59
Cell-Saver^®^ (units ^a^)	1.8 ± 1.8	2.2 ± 2.3	0.08
FFP (units)	4.1 ± 3.8	4.3 ± 3.9	0.77
Platelets concentrate (units)	0.8 ± 0.8	0.7 ± 0.8	0.40

Data are expressed as mean ± standard deviation. PaO_2_, oxygen partial arterial pressure; RBC, packed red blood cells; FFP, fresh frozen plasma. ^a^ One unit is approximately 250 mL of recycled blood.

**Table 4:** Perioperative and postoperative outcomes.

	Group PaO_2_ < 200 mmHg (*n* = 118)	Group PaO_2_ > 200 mmHg (*n* = 104)	*p*
Primary outcome:			
Lactate (mmol/L) < 0.80 15 min after reperfusion	4.81 ± 2	6.03 ± 4	<0.01
Secondary outcomes:			
Lactate end of LT (mmol/L) < 0.80	3.25 ± 2.1	4.22 ± 3.8	0.02
AST peak (IU/L) < 34	1015 ± 1540	1570 ± 2457	0.04
ALT peak (IU/L) < 34	699 ± 856	988 ± 1118	0.03
PR J7 (%)	77.4 ± 16	76.7 ± 20	0.79
Factor V D7 (IU/mL)	1.2 ± 0.38	1.2 ± 0.56	0.75
Bilirubin D7 (µmol/L) < 17 Early allograft dysfunction	86 ± 85 31 (26%)	83 ± 91 43 (42%)	0.80 0.02
Norepinephrine level (mg/h)	1.2 ± 1.4	1.4 ± 1.5	0.34
Duration of MV (hours)	11 ± 13	24 ± 49	<0.01
Duration of ileus (days)	4.3 ± 1.6	4.9 ± 1.9	0.03
ICU length of stay (days)	11.6 ± 14	20.6 ± 48	0.05
Hospital length of stay (days)	27.6 ± 21	32.5 ± 39	0.26
Mortality in ICU	5 (4.2%)	8 (7.7%)	0.27
Mortality at 1 year	12 (10.2%)	14 (13.5%)	0.44
Postoperative complications:			
Acute renal failure	35 (30%)	43 (42%)	0.06
Renal replacement therapy	16 (14%)	17 (17%)	0.54
Hemorrhages	8 (7%)	14 (14%)	0.07
Cardiovascular	18 (15%)	12 (12%)	0.50
Respiratory	11 (9%)	12 (12%)	0.50
Sepsis	32 (27%)	22 (22%)	0.40

Data are expressed as mean ± standard deviation or absolute value (percent). LT, liver transplantation; PR, prothrombine ratio; AST, aspartate amino-transaminase; ALT, alanine amino-transaminase; D7, day 7; EAD, early allograft dysfunction; MV, mechanical ventilation; ICU, intensive care unit.

## Data Availability

Research data are available by request to the corresponding author.

## Abbreviations

LT	liver transplantation
IR	ischemia–reperfusion
ROS	reactive oxygen species
FiO_2_	fraction of inspired oxygen
PaO_2_	oxygen partial arterial pressure
ICU	intensive care unit
PR	prothrombin ration
AST	aspartate amino transaminase
ALT	alanine amino transaminase
AP	alkaline phosphatase
GGT	gamma-glutamyl transferase
MELD	model for end-stage liver disease
NASH	nonalcoholic steatosis hepatitis
HCC	hepatocellular carcinoma
HAA	acute alcoholic hepatitis
MV	mechanical ventilation
RBC	packed red blood cells
FFP	fresh frozen plasma
